# Enriched experience increases reciprocal synaptic connectivity and coding sparsity in higher-order cortex

**DOI:** 10.1101/2025.09.01.673156

**Published:** 2025-09-11

**Authors:** Rajat Saxena, Justin L. Shobe, Aida M. Andujo, Wing Ning, Christelle Anaclet, Bruce L. McNaughton

**Affiliations:** 1 Department of Neurobiology and Behavior, University of California Irvine, Irvine, CA 92697, USA; 2 Canadian Centre for Behavioural Neuroscience, The University of Lethbridge, Lethbridge, AB, T1K 3M4 Canada; 3 Department of Neurological Surgery, University of California Davis, Davis, CA 95616, USA; 4 Present address: Kavli Institute for Systems Neuroscience and Centre for Algorithms in the Cortex, Norwegian University of Science and Technology, Trondheim, Norway

## Abstract

The integration of new information during sleep reshapes cortical representations that support categorical knowledge. Auto-associative attractor network theories predict that reciprocal excitatory connections help form stable categorical attractors, but direct evidence is missing. We tested this using ten weeks of enriched experience (ENR) in mice as a model for knowledge accumulation and recorded single-unit activity across hippocampus and neocortex. ENR induced significant remodeling in high- but not low-level neocortex, with a shift from unidirectional to bidirectional excitatory-excitatory connections, suggestive of increased ‘cell assemblies’. This was accompanied by increased inhibitory-to-excitatory connections and sparser, more orthogonal population activity during awake rest and slow-wave sleep, particularly in deep layers. Thus, ENR reorganizes cortical circuits into a symmetric, inhibition-balanced network that improves coding efficiency, supporting long-standing attractor network predictions.

## Introduction:

In the neocortex (NC), new knowledge is integrated with prior information, which is organized into abstract cognitive schemas or concepts. These schemas enable the flexible recombination of previously learned representations, which facilitates the rapid integration of new information (i.e. forward knowledge transfer). However, the network mechanisms underlying schema storage and organization remain poorly understood.

Hebbian cell assembly theory and modern auto-associative attractor network models, including Hopfield networks, predict that increasing the number of stored patterns leads to more reciprocal (bidirectional) synaptic connectivity (1–4). Such connectivity enhances robustness to noise, enables signal amplification, and supports pattern completion (5). These models also predict that increasing representational sparsity expands memory capacity by reducing overlap between stored patterns – trends consistently observed in artificial neural networks, where both sparsity and orthogonality increase across learning epochs (6).

Here, we tested the hypothesis that environmental enrichment (ENR) (7–9), as a model of knowledge accumulation, would produce neural changes consistent with the foregoing theoretical predictions. Specifically, we predicted that knowledge-rich brains would exhibit increased bidirectional excitatory connectivity, compared to exercise-only control brains. We used a recently developed ENR protocol where animals are exposed to complex, multisensory, and stimulating conditions that enhance both behavioral performance (10) and neural functions (11–13) and combined it with high-density electrophysiology recordings across the retrosplenial cortex, primary visual cortex, and hippocampus (HC). This approach allowed us to examine how enriched experiences alter functional synaptic connectivity and population coding dynamics, particularly during offline periods such as awake rest and slow-wave sleep (SWS), when the brain freely explores its internal state-space (14–17).

### Experimental Paradigm:

**Fig. 1.**
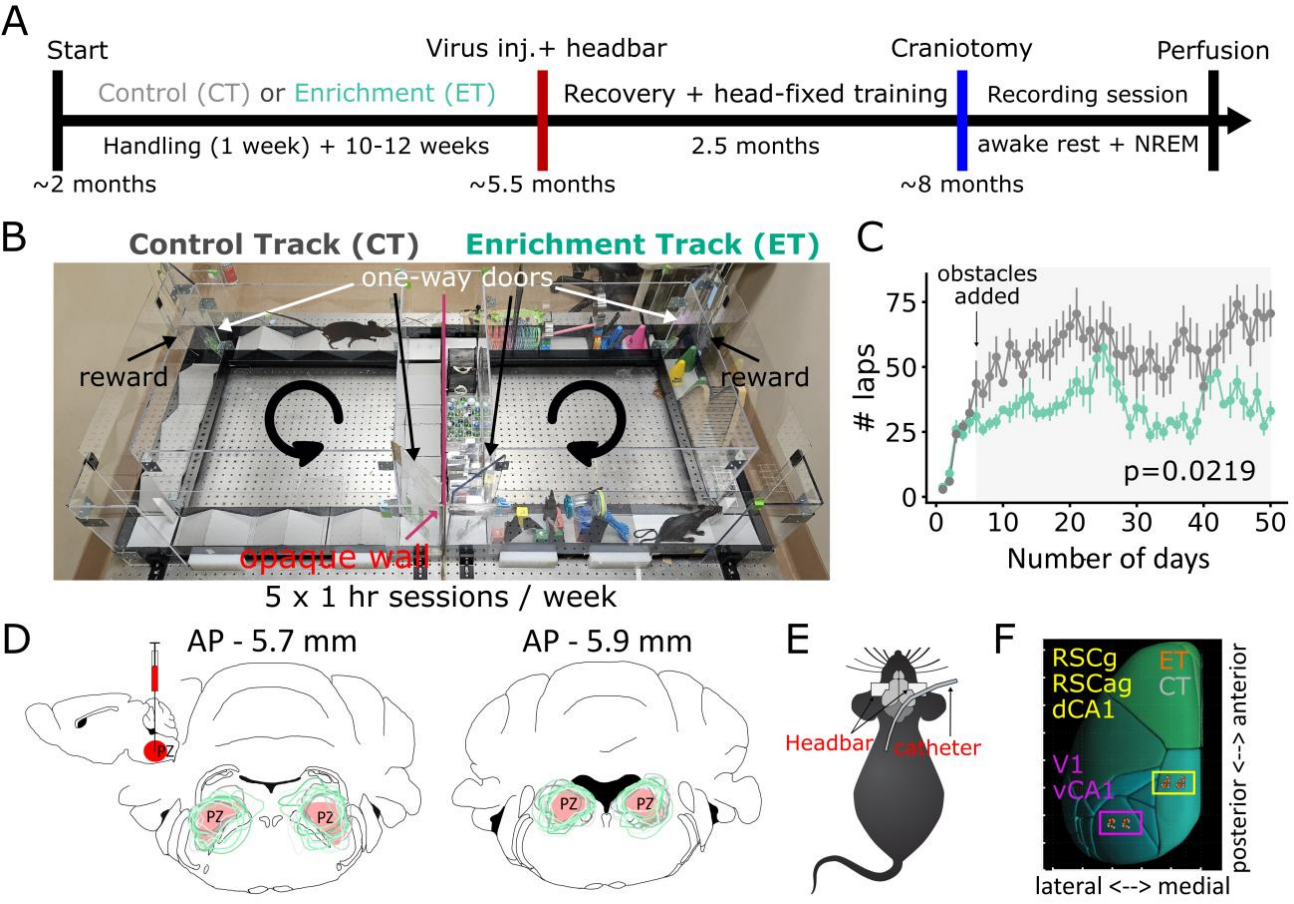
Environmental enrichment (EE) protocol and experimental design (**A**) Timeline: ~2-month-old mice ran for 10–12 weeks on either an enrichment (ET, aquamarine) or control track (CT, gray) (track: 86.5cm long × 86.5cm wide; arm width: 11.43cm). After enrichment/control treatment, animals were head-barred and injected with DREADDs into the Parafacial Zone (PZ) to enable chemogenetic induction of SWS. After ~2–2.5 months of head-fixation training, craniotomies were performed over RSC, V1, and CA1, and a catheter was implanted for subcutaneous CNO delivery. Each mouse underwent one recording session: 2.5–3 hours of awake rest followed by ≥ 13 hours of induced SWS. (**B**) We ran double-housed young adult mice (8 weeks) in our ENR setup such that one mouse ran on CT (left), while the paired cagemate ran on ET (right) for 5 × 1-hour sessions/week for 10–12 weeks. ET mice encountered 12 obstacles (new obstacle configuration every session), while CT mice ran over the same simple ramp hurdles. Arrows indicate running direction. (**C**) Mean number of laps ± sem for 10 weeks (50 days). ET mice ran fewer laps than CT (F=5.938, p=0.033, rmANOVA). (**D**) Inset: schematic showing pAAV2-hSyn-DIO-hM3D(Gq)-mCherry injected (bilaterally) into the PZ (red). Virus expression for both ET (green) and CT mice (gray) for two coronal sections. Schematic of (**E**) catheter implant and (**F**) dual-probe craniotomies: dCA1, DG, RSCag, RSCg (yellow box); V1, vCA1 (pink box). Colored circles (ET=orange and CT=gray) mark each animal’s recording sites for both probes (mirrored for recording from other hemisphere).

We ran n=18 (2-month-old, 10M/8F) pair-housed mice on either an enrichment track (ET; n=5M/4F) or an exercise control track (CT; n=5M/4F) for 10–12 weeks (5 × 1-hour sessions per week) (Fig. 1A-C). ET mice were exposed to multiple obstacles that were changed daily to allow a rich repertoire of visual, somatosensory, and motor experiences, while CT mice ran on a track with simple ramp hurdles (20 cm × 11 cm per hurdle) (Fig. S1A, B). CT mice ran more laps than ET mice (F=5.938, p=0.033, group-effect, repeated-measures ANOVA (rmANOVA)) (Fig. 1C). A separate group of n=15 mice (7ET/8CT, age-matched to main group) underwent the same ENR protocol and confirmed that ET group showed enhanced spatial recognition in a Y-maze task (t=−4.2, p=0.0013, unpaired t-test; Fig. S1C-F), replicating previously shown behavioral benefits of ENR using this paradigm (10).

After ENR, mice (~5.5 months old) were head-barred and injected bilaterally with pAAV2-hSyn-DIO-hM3D(Gq)-mCherry virus in the parafacial zone (PZ), a SWS promoting region, to induce essentially natural SWS at will using Clozapine-N-oxide (CNO) (18) (Fig. 1D). This allowed stable data collection for long-duration offline periods, which is required for reliable estimation of connections between excitatory neurons (19). After ~2 months of head-fixation habituation, craniotomies were performed over granular and agranular retrosplenial cortex (RSCg, RSCag), primary visual cortex (V1), dentate gyrus (DG), and dorsal and ventral CA1 (dCA1, vCA1), and a catheter was implanted in the upper back for subcutaneous CNO injection (Fig. 1E-F).

Acute electrophysiology recordings were conducted the day after craniotomy surgery using two dual-shank 256-channel silicon probes (512 total) (20), targeting RSC-dCA1 (probe 1) and V1-vCA1 (probe 2). Recordings included 2.5–3 hours of awake rest, followed by at least 13 hours of CNO-induced slow-wave sleep (SWS), with CNO boosters administered subcutaneously (0.5 mg/kg), every 2.75±0.12 hours. Average recording duration was 2.85±0.06 hours (awake rest) and 17.67±0.44 hours (SWS). Brains were then sectioned to verify recording locations (Fig. 1F; Fig. S2A; Table S1) and virus expression, which did not differ across groups (t=0.276, p>0.05, unpaired t-test; Fig. 1D; Fig. S2B). Recordings were randomly interleaved between groups as the experimenters were blind to the group identity.

**Fig. 2.**
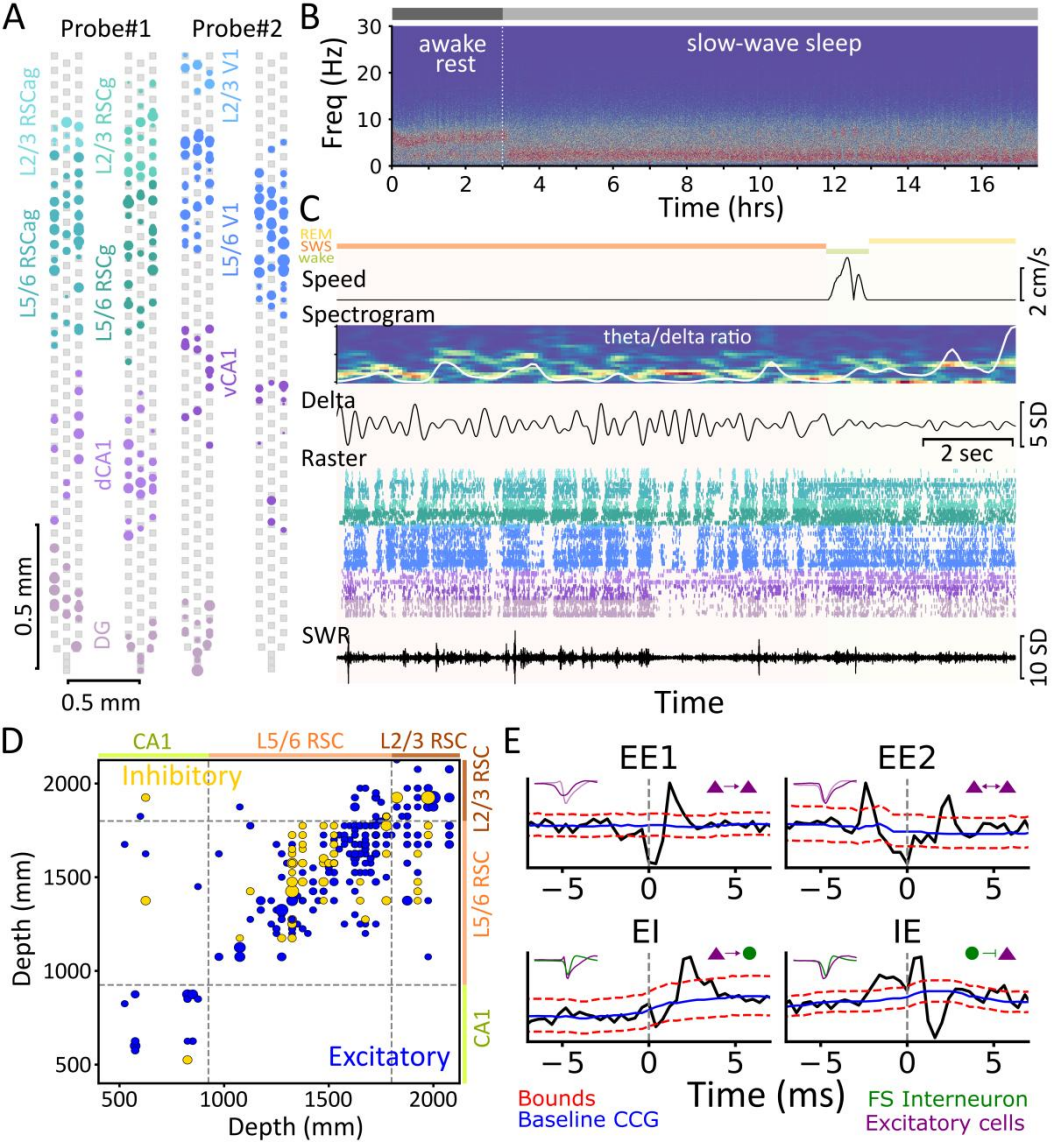
High-density electrophysiology recordings and monosynaptic connectivity analysis (**A**) Left: 256-channel probe (gray squares mark recording sites spanning 2.125 mm) targeting DG (lily), dCA1 (lavender), L5/6 RSCg (sea green), L2/3 RSCg (pale teal), L5/6 RSCag (fountain blue), and L2/3 RSCag (aquamarine blue) from an example animal. Right: second probe targeting L2/3 V1 (light sky blue), L5/6 V1 (dark sky blue) and vCA1 (lilac) (L4 units excluded). Circles show single units; size reflects mean firing rate. (**B**) Spectrogram from an L5 RSCag channel showing 3 hours of awake rest, followed by ~17.5 hours of induced SWS. (**C**) Brain states: SWS (orange), REM (yellow), and wake (green) are on top. Zoom-in on a 15 s segment showing speed (cm/s), spectrogram (0–20 Hz) with theta/delta ratio (white line) overlaid, delta-filtered (1–4 Hz) signal from the same channel as (B), spike raster sorted by region and depth, and sharp-wave ripple (SWR, 110–250 Hz, middle) filtered trace from dCA1. Same color scheme as (A) is used for spike raster. (**D**) Putative excitatory (blue, include EE and EI) and inhibitory (gold) connections across regions and layers (dCA1, L5/6 RSCg/RSCag, and L2/3 RSCg/RSCag) for a representative animal. Dot size indicates synaptic strength. In dCA1, excitatory connections observed are excitatory-to-inhibitory (EI) connections only. (**E**) Example Cross-correlogram (CCG) example showing unidirectional excitatory-excitatory (EE1), bidirectional excitatory-excitatory (EE2), excitatory-inhibitory (EI), and inhibitory-excitatory (IE). Excitatory units (E) and fast-spiking interneurons (I) are shown as purple triangles and green circles, respectively. Inset: waveforms of pre- and post-synaptic units. Solid blue line: baseline; red dashed line: confidence intervals (α=0.001).

Spike sorting (21) yielded single units (CT: 176.86±11.82, 124.0±38.28; ET: 206.0±20.58, 118.72±36.85 for probe1 and probe2, respectively; Fig. 2A, Table S1). Putative excitatory (mean firing rate: 2.523±0.049 Hz) and fast-spiking (FS) interneurons (mean firing rate: 10.038±0.359 Hz) were classified based on the waveform and auto-correlogram features (Fig. S2D). Four animals were excluded due to low cell yield or surgical issues, resulting in a final sample of n=14 animals (CT: 7 (4M/3F); ET: 7 (4M/3F)) for the RSC probe and n=10 animals (CT: 5 (3M/2F); ET: 5 (3M/2F)) for the V1 probe.

Brain state (wake, SWS/NREM, REM) was scored using running speed, theta (5–10 Hz), and delta (1–4 Hz) oscillation power (Fig. 2B-C) (22). ET group showed slightly reduced delta power in the last 2–3 hours (F=1.741, p=0.023, group*time effect, rmANOVA) (Fig. S3B). Therefore, we analyzed only the first 15 hours (~12 hours SWS). No group differences were found in total awake: CT: 2.56±0.12, ET: 2.6±0.11 hours (t=−0.185, p=0.85, unpaired t-test), SWS duration: CT: 9.9±0.22, ET: 10.1±0.2 hours (t=0.688, p=0.504; Table S1), SWS proportion (F=0.007, p=0.935; Fig. S3C), SWR rate (F=0.045, p=0.835, group-effect rmANOVA; Fig. S3C1), and other SWR-related properties (p>0.05, unpaired t-test with Bonferroni correction; Fig. S3C2–4).

### Changes in functional monosynaptic connectivity:

We first analyzed functional synaptic connectivity in NC to test whether enriched experiences remodel NC circuitry to support encoding of large number of experiences. We computed cross-correlogram (CCG) for all simultaneously recorded neuron pairs and identified short-latency peaks or troughs (0.8–4.8 ms) to identify putative monosynaptic excitatory or inhibitory connections (19, 23) (Fig. 2D). While we refer to these connections as functional, their validity is supported by juxtacellular recordings, which have demonstrated that such CCG peaks and troughs reflect true monosynaptic connections (24). Each significant connection was further classified as unidirectional excitatory-excitatory (EE1), bidirectional excitatory-excitatory (EE2), excitatory-inhibitory (EI), and inhibitory-excitatory (IE), based on short-latency peak/trough and cell classification (Fig. 2E). Common input connections with zero-lag synchrony, typically observed between interneuron pairs (II), were excluded (24).

**Fig. 3:**
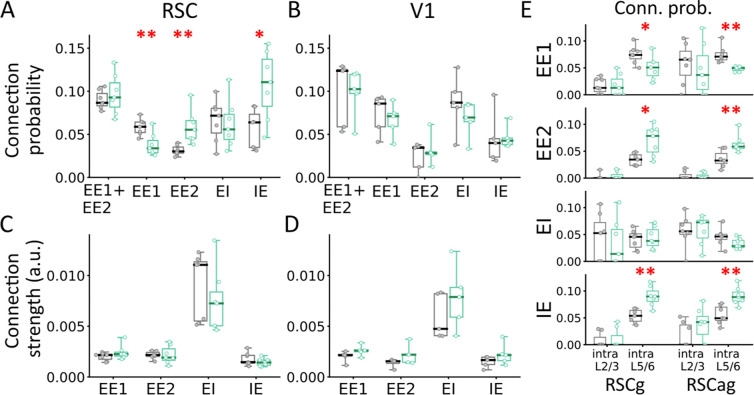
Enriched experiences alter functional monosynaptic connectivity in deep RSC. Connection probability for all excitatory-to-excitatory (EE1+EE2), EE1, EE2, and IE connections for (**A**) RSC (n=7CT/ 7 ET) and (**B**) V1 (n=5CT/ 5ET) for ET (aquamarine) and CT (gray) mice. Horizontal solid lines indicate the distribution mean. A similar plot is shown for synaptic interaction strength (a.u.) calculated from sum of values above or below chance CCG in the 0.8–4.8 ms range for (**C**) RSC and (**D**) V1 for both groups. (**E**) Connection probability for within-layers (intralaminar) EE1, EE2, EI, and IE connections (top to bottom) for RSCg and RSCag. P-values for unpaired t-test are listed at the top of each combination. *p<0.05, **p<0.01.

We analyzed a total of 7,611.43±955.98 excitatory (EE1+EE2) and 3,818.43±525.23 inhibitory (EI+IE) cell pairs in RSC (n=7CT/7ET), and 3,376.5±710.25 excitatory and 1,880.5±344.81 inhibitory pairs in V1 (n=5CT/5ET). The total number of excitatory-to-excitatory (EE1+EE2) connections were comparable between groups in both RSC and V1. However, ET mice showed a significant increase in EE2 connections (t=−3.563, p=0.009; unpaired t-test; Table S2) and IE connections (t=−2.933, p=0.0158; Table S2) specifically in RSC, but not in V1 (Fig. 3A,B). This increase was accompanied by a significant decrease in EE1 connections in RSC (t=3.361, p=0.006; Table S2). No difference was observed in connection probability for EI connections.

Next, we assessed synaptic connection strength using: 1) sum of observed CCG values exceeding (or below) chance, normalized by the reference cell’s spike count, 2) z-score peak (or trough) relative to the chance-level CCG. For EE2 connections, strength was averaged across both sides of the CCG. No significant group differences in connection strength were observed for any connection type in either region (Fig. 3C,D; Table S2; Fig. S4A). Consistent with previous studies, connection probability declined with distance (Fig. S4B). EI connections were generally stronger than EE1 and EE2 connections (Fig. S4B), and all connection types showed lognormal strength distributions (Fig. S4C) (23, 25, 26). Unlike a previous study (23), we did not observe stronger EE2 connections relative to EE1 (Table S2). No sex differences were observed (p>0.05, ANOVA with Bonferroni correction, Fig. S7A).

The expected number of unconnected, EE1, and EE2 pairs was estimated using the following equations: N(1-p)^2^, 2Np(1-p), and Np^2^, respectively, where N is the total EE pairs and p is the overall EE connection probability (19, 23). Using p=0.1 (p_EE1_ + p_EE2_; Fig. 3A,B) and the observed N, we found that the number of EE1 connections were lower than expected, whereas EE2 were significantly higher (~3x) than expected (p<0.05). Similar increase in EE2 connections have been reported previously (19, 27). This overrepresentation of bidirectional EE2 (symmetric) connections is consistent with attractor dynamics and may reflect circuit-level changes supporting the encoding of multiple items experienced during ENR (5).

In V1, we analyzed intralaminar deep (L5/6) connections in Fig. 3B,D, as insufficient number of superficial (L2/3) neurons were available across animals. In contrast, RSC data included both L2/3 and L5/6 neurons from granular (RSCg) and agranular (RSCag) subdivisions. Therefore, we decided to analyze connections within and between L2/3 and L5/6of RSCg and RSCag. Interareal connections were rare, but we detected some interlaminar connections within the same brain region for EE1 (probability in RSCg: CT: 0.011±0.003, ET: 0.012±0.004; RSCag: CT: 0.025±0.004, ET: 0.022±0.005), EI (RSCg: CT: 0.015±0.004, ET: 0.016±0.006; RSCag: CT: 0.020±0.005, ET: 0.020±0.007), and IE (RSCg: CT: 0.019±0.004, ET: 0.016±0.004; RSCag: CT: 0.008±0.004, ET: 0.010±0.005) connection types, but not for EE2, with no significant group differences in their probability or strength. Thus, we focused on intralaminar connections within each RSC subregion.

The total number of intralaminar EE pairs were: L2/3 RSCg: 91.08±17.89; L5/6 RSCg: 1194.0±211.01; L2/3 RSCag: 82.64±10.56; L5/6 RSCag: 1417.21±176.36 and EI/IE pairs were: L2/3 RSCg: 71.63±11.5; L5/6 RSCg: 620.93±118.3; L2/3 RSCag: 70.69±13.44; L5/6 RSCag: 454.21±70.48. In ET mice, EE2 connection probability was significantly higher in deep (L5/6) layers of both RSCg (t=−3.149, p=0.013) and RSCag (t=−3.177, p=0.008) compared to CT mice (Fig. 3E). In contrast, the EE1 connection probability was significantly lower in ET mice in L5/6 of both RSCg (t=2.36, p=0.037) and RSCag (t=4.259, p=0.003), consistent with Fig. 3A results. EI connection rates were unchanged across groups, but IE connection probability was significantly increased in ET mice in L5/6 of both RSCg (t=−4.337, p=0.0013) and RSCag (t=−3.894, p=0.002) (Fig. 3E). Across animals, L2/3 connection probabilities were often zero, suggesting that the results in Fig. 3A are largely driven by L5/6 neurons. Finally, no group differences were observed in intralaminar synaptic strength across connection types in either RSCg or RSCag (Fig. S4D). Subsampling L5/6 neurons to match the number of L2/3 neurons yielded similar results, ruling out sampling bias.

We computed CCG over the entire 15-hour recording to obtain a reliable estimate of connection probability (19). However, awake rest and SWS differ in several aspects, including activity levels, neuromodulatory tone, memory replay dynamics, etc. (28–31). To assess state-dependent changes, we recalculated connection strength for connections identified in the full recording, separately for awake rest (~2.5–3 hours) and the first 3 hours of SWS. Previous studies reported that intralaminar L5/6 EE connection strength increases from awake rest to SWS (25). We also observed the largest differences in intralaminar L5/6 EE and IE connection probabilities (Fig. 3A,B). We therefore focused our analysis on intralaminar L5/6 connections to determine whether similar changes in connection strength occur. No significant group differences were found; although EI, IE, and EE2 connections showed a slight upward trend from awake rest to SWS in both groups (Fig. S4E), while EE1 remained stable. Together, these results highlight selective reorganization of deep-layer RSC connectivity following EE, characterized by a shift toward more reciprocal (bidirectional) excitatory connections and enhanced inhibitory feedback, consistent with attractor-like dynamics.

### Changes in population coding dynamics:

Having established the changes in synaptic connectivity, we next asked whether ET mice would show sparser and more orthogonal population activity. We computed population and lifetime sparseness using 50 ms binned population vectors (PV) of excitatory units during awake rest and SWS. Lifetime sparseness reflects how selectively a single neuron fires over time, while population sparseness quantifies the proportion of active neurons in a population at any given time (32, 33). Neuron counts were balanced across animals and brain regions by sub-sampling 1000 times and averaging. We compared units from CA1, L2/3 RSC, and L5/6 RSC separately due to their distinct coding properties (34–36). For V1, we focused on L5/6 neurons due to insufficient L2/3 neurons.

**Fig. 4:**
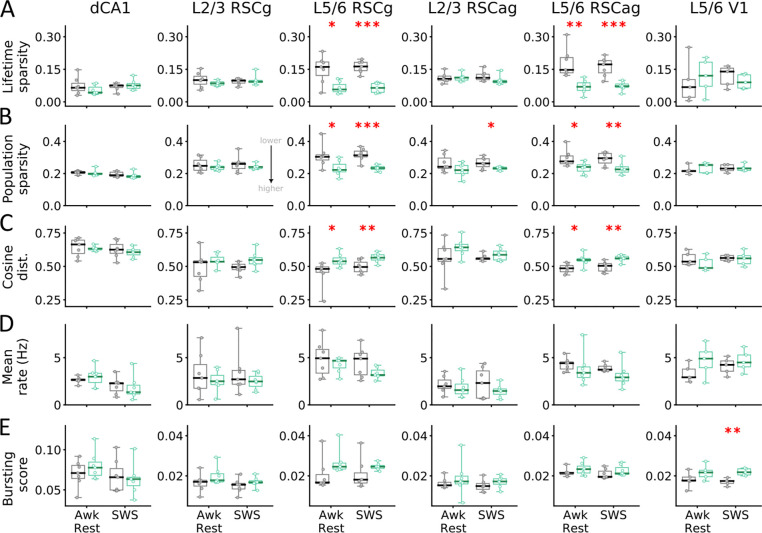
Enriched experiences increase sparsity and orthogonality in RSC deep layers. Changes in (**A**) lifetime sparsity, (**B**) population sparsity, (**C**) cosine distance, (**D**) mean firing rate (Hz), and (**E**) bursting score for excitatory neurons across brain regions: dCA1, superficial (L2/3) and deep (L5/6) granular and agranular retrosplenial cortex (RSCg/RSCag) and primary visual cortex (V1) for ET (aquamarine) and CT (gray) mice during awake rest and SWS. Lower values indicate more sparsity (A,B). Each point represents data from one animal. Total n=14 mice (7CT/7ET) for RSC and dCA1, and n=10 (5CT/5ET) for V1. ET mice show higher lifetime, population sparsity, and cosine distance for L5/6 RSC ensembles, but not for V1. *p<0.05, **p<0.01, ***p<0.001.

During both awake rest and SWS, ET mice showed significantly increased population and lifetime sparseness in L5/6 RSCg (lifetime sparsity: Awk Rest: t=−3.147, p=0.012; SWS: t=−6.628, p<0.001; population sparsity: Awk Rest: t=−3.23, p=0.008; SWS: t=−4.897, p<0.001) and L5/6 RSCag (lifetime sparsity: Awk Rest: t=−3.812, p=0.004; SWS: t=−4.8, p<0.001; population sparsity: Awk Rest: t=−2.36, p=0.038; SWS: t=−3.15, p=0.009) (Fig. 4A,B; Table S3). A modest but significant increase in population sparseness was also observed in L2/3 RSCag in ET mice during SWS (t=−2.445, p=0.043; unpaired t-test). No group differences in sparseness were found in other regions (Fig. 4B, Table S3). Dividing the 12-hour SWS into 3-hour blocks showed comparable results, with ET mice exhibiting increased sparsity in RSC L5/6 neurons (Fig. S5A,B). As no systematic differences were observed across SWS blocks, we report data pooled across the entire SWS period (Fig. 4).

We then looked at orthogonality across brain regions using average cosine distance between pairs of PVs. ET mice exhibited significantly larger distances in L5/6 RSCg (Awk Rest: t=−2.816, p=0.017; SWS: t=−3.15, p=0.009) and L5/6 RSCag neurons (Awk Rest: t=−2.725, p=0.023; SWS: t=−3.464, p=0.005) (Fig. 4C, S5C; Table S3), indicating a more diverse neural state space. Other regions did not show significant group differences. Increase in sparsity and orthogonality were generally more pronounced during SWS, potentially reflecting differences in activity levels and memory replay dynamics between the two states (28–31). Perhaps a more diverse activity space is being explored during SWS than awake rest, which might be more prominent in ET mice. Differences in sparsity and orthogonality could not be fully explained by differences in mean firing rate or bursting index (proportion of spikes with inter-spike interval < 6 ms), as no significant group effects were detected for either measure (Fig. 4E,F, Fig. S5, Table S3). That said, a trend toward more bursting and lower firing rates in ET mice was visible. Consistent with previous studies, mean firing rates were generally higher in NC L5/6 compared to L2/3 (34–36) and lower during SWS than awake rest (37, 38). No significant sex differences were found (p>0.05, ANOVA with Bonferroni correction, Fig. S7b).

Changes in population and lifetime sparsity were primarily observed in L5/6 RSC. The lack of significant changes in CA1 and L2/3 RSC may be attributed to these regions already operating at near-maximal (or near-optimal) sparsity levels, whereas L5/6 neurons may have greater potential for improving coding efficiency (34, 35). No significant difference in lifetime sparsity was observed in DG neurons after pooling data across animals (Fig. S6A). This finding, combined with the lack of difference in CA1 sparsity, contrasts with previous studies examining hippocampal subregions after ENR (39–41). As CT mice ran more laps (Fig. 1C), more physical exercise may have promoted neurogenesis and hippocampal sparsity, potentially reducing differences between the groups (42, 43). L2/3 RSC likely follows a similar pattern, as it receives direct inputs from dCA1 (44, 45). However, a more likely explanation is that L5/6 neurons have been shown to code for categorical knowledge (46, 47). Consequently, larger effects are expected in L5/6 of ET mice to accommodate a greater diversity of sensory-motor experiences. Selective increase in L5/6 RSC, but not in L5/6 V1, may result from V1 being an early sensory area where feature coding is relatively less experience-dependent. Alternatively, RSC integrates multimodal inputs from multiple areas beyond V1, such as somatosensory and motor, which may exhibit more pronounced changes and drive the observed results (48). Supporting this, a recent study using the same ENR paradigm demonstrated rapid stabilization of spatially selective firing in secondary motor cortex, a primary input region to RSC (11). Further exploration of PV correlations using graph-based analysis trends towards lower clustering coefficient (not significant) for L5/6 units in ET mice, indicative of weaker coactivation, in alignment with higher orthogonality observed (Fig. S6C).

## Discussion:

The most significant finding of this study is the *in vivo* validation of a key prediction from auto-associative attractor networks: that knowledge accumulation should lead to an increase in reciprocal (bidirectional) excitatory-excitatory connections (1–4). Our results demonstrate that enriched experience, used here as a model for knowledge accumulation, produces a substantial shift from unidirectional to bidirectional excitatory-excitatory connections in higher-order cortex, providing key experimental evidence for this long-theorized mechanism of cortical rewiring. This finding has important implications for our understanding of how the brain stores and organizes knowledge. Attractor network models predict that reciprocal synaptic connectivity enhances robustness, enables signal amplification, and supports pattern completion (5), all critical functions for maintaining stable memory representations. The observed shift toward more symmetric excitatory connections in ET mice, exposed to a wide range of experiences, directly supports these theoretical predictions and suggests that enriched brains develop connectivity patterns optimized for storing multiple, stable attractor states.

Complementing this primary finding, we observed that ENR also enhanced representational sparsity and orthogonality in population codes, particularly in deep RSC layers. ET mice exhibited significantly increased population and lifetime sparseness in L5/6 RSC during both awake rest and slow-wave sleep, accompanied by larger cosine distances between population activity. We also found a trend toward increased bursting and a reduced mean firing rate in L5/6 RSC (though not significant), suggesting that their combined effect may contribute to the observed changes in coding sparsity. These changes in sparsity align with network simulations, which show that increased sparsity and bidirectionally connected excitatory pairs characterize systems optimized to maximize storage capacity through fixed attractors (23, 49–51). Together, the convergence of our connectivity and population coding results provides compelling evidence that ENR reorganizes cortical circuits according to principles predicted by computational theories of memory storage.

The observed increase in inhibitory-to-excitatory connections could serve to balance the high gain resulting from more bidirectional excitatory connections, thereby preventing runaway excitation. Alternatively, consistent with modeling work showing that enhanced inhibition promotes the formation of non-overlapping cell assemblies (52), increased inhibition in ET mice may facilitate the development of orthogonal cell assemblies, reflected in the prevalence of bidirectional excitatory connections and greater sparsity. The sparser and more orthogonal representations we documented may promote faster learning by minimizing overlap between representations, thereby reducing interference with existing memories (53–55). This could contribute to the enhanced behavioral and forward knowledge transfer consistently observed in enriched animals (8, 10, 11).

Several limitations should be acknowledged. We used a specific ENR paradigm (10) that significantly improves behavioral performance compared to standard protocols, but our findings may not generalize across all enrichment paradigms. The chemogenetic enhancement of SWS (18, 56), while enabling long, stable recordings necessary for detecting weak excitatory connections (19), disrupted normal sleep cycles during recording sessions (which were conducted long after the enrichment treatment itself). Future work should examine how connectivity changes evolve over the course of enrichment protocol. In addition, assessing whether comparable changes occur in other cortical regions, such as prefrontal, motor, or somatosensory cortex would help establish the generality of our findings. It would also be interesting to compare higher-order visual and somatosensory cortex in modality-specific enrichment, i.e., visual-only or somatosensory-only.

In summary, our findings provide evidence supporting cell assembly (1) and modern attractor network predictions, including Hopfield networks, (2–4) regarding cortical connectivity changes following knowledge accumulation. The observed shift toward enhanced bidirectional excitatory and inhibitory connections, accompanied by sparser and more orthogonal population representations, suggests that enriched experiences reorganize cortical circuits to optimize memory storage and retrieval. These results link theoretical predictions from computational neuroscience with empirical observations, advancing our understanding of how lifelong learning shapes brain connectivity and function.

## Supplementary Material

Supplement 1

## Data Availability

All processed data and analysis scripts will be made public before publication.
